# Red flags in neuro-ophthalmology

**Published:** 2017-03-03

**Authors:** Olufunmilola Ogun

**Affiliations:** 1 Lecturer and Honorary Consultant, Department of Ophthalmology, College of Medicine, University of Ibadan and University College Hospital Ibadan, Nigeria.

**Some diseases of the brain presenting with visual symptoms are life threatening and need urgent management. This article discusses possible causes of double vision, vision loss with headache, and non-ocular vision loss.**

**Figure:**
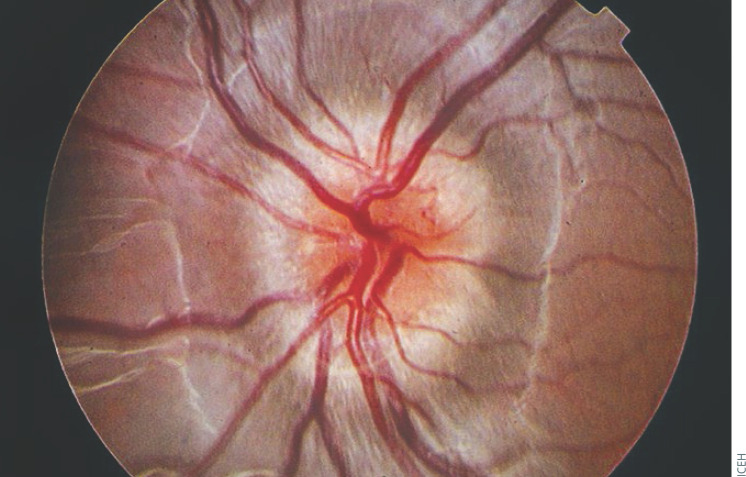
Papilloedema in a 23-year-old woman. Headache for two months. Worse on waking. Vision 6/6 in both eyes.

The three most important ‘red flag’ symptoms that indicate that a patient may need neuro-ophthalmological assessment are:

1. Sudden onset of double vision (diplopia)
2. Headache accompanied by vision loss (without an ocular cause)
3. Visual loss after ocular causes have been excluded

If a patient presents with any of the symptoms above, you must take a detailed history (**Table 1**).

## 1 Sudden onset of double vision

**Figure 1.**
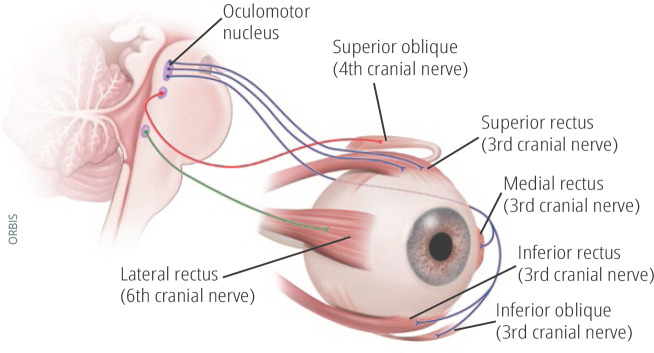
The three cranial nerves

Each eye is moved by six muscles which are innervated by three “cranial” nerves (the 3rd, 4th and 6th nerve) (**Figure 1**). If the nerves are affected then the eye cannot move normally, which results in double vision. The 3rd nerve also innervates the upper eyelid (**Table 2**).

First exclude monocular diplopia by asking the patient to cover each eye in turn. If the double vision persists when looking with just one eye, then this is usually due to an ocular problem (e.g. cataract) and does not have a neurological cause.

**Table 1:** Taking a history

Questions to ask	Clinical interpretation
Is the double vision worse in any direction of gaze?	The direction of gaze in which the double vision is worst signifies the most likely eye muscle involved.
Are the images side by side; or is one image tilted and above the other?	In 6th nerve palsies the images are side by side; In 4th nerve palsies one image is tilted.
Has there been a recent head injury?	Trauma to the brain or orbit can affect the nerves which control eye muscle movements.
Does the double vision get worse as the day progresses or after exercise?	If the condition gets worse with use of the muscle then this is typical for myasthenia gravis; there may be eyelid drooping (ptosis) or diplopia as the day proceeds.
Is there any head or eye pain?	Pain is an important clue: it usually indicates infection or inflammation. Tumours are less likely to be painful.
Are there any systemic symptoms or diseases?	Hypertension and diabetes can both cause loss of vision and diplopia.

**Table 2:** Examination

Questions to ask	Clinical interpretation
Is the upper eyelid drooping?	Ptosis may be due to myasthenia gravis or a third nerve palsy, or may be congenital.
How do the eyes move (each eye alone and both eyes together)?	Assess the position of the eyes looking straight ahead (check for squint), and the movements of each eye alone and together in all 9 positions of gaze (**Figure 3** on page 67). Limitation of movement in a certain direction indicates disease of the affected muscle or the cranial nerve which innervates it. 6th nerve palsy: eye cannot look out (abduction). 3rd nerve palsy ptosis and eye cannot look up and in.
Are the pupils of equal size?	If one pupil is larger than the other, this suggests 3rd nerve palsy relating to the eye with the larger pupil.
Does the pupil react normally to light?	A non-reactive pupil indicates a damaged optic nerve or prior use of dilating drops.

### What should I do?

Refer all patients with double vision for further investigation. Some may have life-threatening conditions.

## 2 Headache accompanied by vision loss (without an ocular cause)

The brain is encased by the skull and meninges and is bathed in cerebrospinal fluid. If the flow of fluid is blocked, by a tumour for example, this raises the pressure inside the head (intracranial pressure), causing headache, sometimes with nausea or vomiting. Raised intracranial pressure can lead to swelling of the optic nerve head (papilloedema), usually in both eyes. If the raised pressure persists, the optic nerves become atrophie; i.e. they become paler then normal.

Both headaches and visual loss are common. Before suspecting a neurological cause, examine the patient to exclude eye conditions which might be responsible for the visual loss (**Table 4**).

### Taking a history

Ask questions about any aches or pain using the mnemonic **‘SOCRATES’** (**Table 3**).

**Table 3:** Structured history

Questions to ask	Clinical interpretation
**S**ite: Where is the headache? The patient may describe or point to the location	Pain overlying the sinuses may suggest sinusitis, whereas periorbital pain suggests orbital pathology.
**O**nset: How long have they had the headache? Is It worse at any time of the day?	Migraine is a common cause of recurrent severe headache which may last hours or even days. Pain which Is worse In the morning on waking up may be due to raised Intracranial pressure. The headache may be associated with nausea and vomiting.
**C**haracter: Can they describe the quality or type of pain?	Dull, constant, unrelieved pain over days or weeks may suggest a space-occupying lesion. A sudden, throbbing pain Is more typical of vascular problems like migraine or an aneurysm.
**R**adiation: does the pain start In one place and then extend/spread to another?	Pain that starts In one place and seems to move or ‘radiate’ to another suggests that Is generated by the Irritation of a nerve.
**A**ssociated vision loss	Severe constant headache with gradual visual loss suggests either compression of the optic nerve, or longstanding raised Intracranial pressure.
**T**ime course: have the symptoms changed over time?	Symptoms which are constant and getting more severe may Indicate a serious progressive condition e.g. a tumour. Intermittent headaches are more typical of vascular or Inflammatory conditions.
**E**xacerbating factors: What makes the headache worse or better?	A headache which Is worse when lying down or bending down may be due to raised Intracranial pressure.
**S**everity: Ask the patient to rate the severity on a scale from 1 (mild) to 10 (very severe)	Any headache that Interferes with the patient's dally activities should not be Ignored.

**Table 4:** Examination

Questions to ask	Clinical interpretation
Are the pupils of equal size and do they react normally to light?	See Table 2.
Is there optic disc swelling or atrophy?	Swelling of the optic disc can be due to raised intracranial pressure (papilloedema) or inflammation (papilitis). Optic atrophy may be due to longstanding compression of the optic nerve or vascular or toxic damage to the nerve.

### What should I do?

Refer all patients with headache and persistent visual loss for further investigation. The referral must be urgent if they have papilloedema. Some may have life-threatening conditions.

## 3 Visual loss after ocular causes have been excluded

Most causes of visual loss are due to diseases of the eye. Ocular conditions must be excluded by careful examination of the eye before considering a neurological cause of poor vision (**Table 5**).

Progressive vision loss with no ocular cause must be taken seriously.

**Table 5:** Visual loss

Questions to ask	Clinical interpretation
Is the vision loss in one or both eyes?	Unilateral vision loss indicates a problem within the eyeball or optic nerve in the orbit.
Has there been any change in the vision since onset?	Vision loss that is progressively worsening may suggest a space-occupying lesion.
Are there any other symptoms?	Vomiting, seizures, and changes in mood or mental state may indicate increased intracranial pressure. Calll for URGENT referral.
Is there a fever?	Fever indicates infection, check the sinuses, ears, orbit, and for neck stiffness.

**More red flags**

**Proptosis**

Proptosis is anterior displacement of the globe. It may be due to space-occupying lesions in the orbit. Adults with acquired proptosis need to be evaluated for thyroid disorder. Pulsatile proptosis, painful proptosis and all cases of proptosis associated with vision loss should be referred for urgent evaluation.

**Ptosis**

Drooping of the upper lid is called ptosis. All cases of acquired ptosis should be evaluated by an ophthalmologist. Marked unilateral ptosis with ocular deviation down and out are signs of a 3rd cranial nerve palsy. If associated with severe sudden onset of unilateral headache this can be due to a intracranial aneurysm (dilated artery). Patients must be referred for immediate neuro-ophthalmological review.

Bilateral ptosis which gets worse as the day progresses may be due to myasthenia gravis.

Partial ptosis with a smaller (constricted) pupil on the same side is due to damage to the sympathetic nerves which supply the muscles in the eyelid and iris – this is called Homers syndrome and the cause needs to be investigated.

